# Difficulty in diagnosing mild cases of COVID‐19 without respiratory symptoms during the Novel Coronavirus Pandemic: Careful monitoring needed for patients with persistent upper gastrointestinal symptoms

**DOI:** 10.1002/ccr3.3248

**Published:** 2020-08-31

**Authors:** Hiroaki Saito, Akihiko Ozaki, Yasuhiro Mizuno, Kozo Todo

**Affiliations:** ^1^ Department of Gastroenterology Sendai Kousei Hospital Sendai Japan; ^2^ Department of Breast surgery Jyoban Hospital of Tokiwa Foundation Iwaki Japan; ^3^ Marru‐clinic Yokosuka Yokosuka Japan; ^4^ Todo Oral and Maxillofacial Surgery Clinic Takaishi Japan

**Keywords:** COVID‐19, FD, gastrointestinal symptoms, GERD

## Abstract

Gastrointestinal symptoms have been reported to occur with COVID infection, and clinicians in COVID‐19‐endemic areas should suspect COVID‐19 infection in patients even if they have no noticeable respiratory symptoms and only gastrointestinal symptoms.

## Graphical abstract

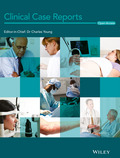

## 1 INTRODUCTION

A 27‐year‐old healthy man presented with sore throat and persistent upper gastrointestinal symptoms for approximately two months and finally turn out to be infected with COVID‐19. Clinicians in COVID‐19‐endemic areas should suspect COVID‐19 infection in patients even if they have no noticeable respiratory symptoms and only gastrointestinal symptoms.

Ever since the first infection cluster was detected in China in December 2019,^1^ the spread of Coronavirus Disease 2019 (COVID‐19) has progressed to the level of a global pandemic. The mortality rate for COVID‐19 is estimated to be 2.0%‐4.4%.^2^ Although this rate is thought likely to be even lower when yet‐undiagnosed cases are included, certain groups such as the elderly and people with complications (eg, diabetes and hypertension) are known to exhibit a high incidence of serious cases.^1^, ^2^ As such, during the spread of COVID‐19 in different regions, diagnosing infected persons is important to prevent further spread of the infection. Different methods are employed to isolate and diagnose infected people, including polymerase chain reaction (PCR) testing, and serum antibody tests are being used together with clinical assessment of symptoms. In addition, various measures have been taken to facilitate rapid diagnosis and infection prevention, including drive‐through testing,^3^ and contact tracing people who have come close to an infected person through the use of mobile devices,^4^ and evaluated for the efficacy.

The most common symptoms of COVID‐19 in the early stages of infection are fever and respiratory symptoms.^2^ However, details on nonrespiratory symptoms have also recently emerged. For example, it is now known that infected people can develop gastrointestinal symptoms such as diarrhea and nausea, headaches, appetite loss, and muscle pain.^1^, ^2^ These symptoms generally manifest in both COVID‐19 and the common cold, which have been frequently observed in outpatients. Discerning which cases are COVID‐19 infections based on these symptoms has proven to be difficult. Moreover, the number of reports and the extent of consideration of actual clinical issues that arise when treating COVID‐19 cases without respiratory symptoms are still insufficient. In light of this, we present a case for COVID‐19 diagnosis based on gastrointestinal symptoms. The written informed consent was obtained from the patient.

## 2 CASE REPORT

A 27‐year‐old healthy man who lived in a major urban area in Japan admitted to our hospital with complaints of sore throat and abdominal discomfort and diminished appetite persisting for 2 months. Sore throat and nasal congestion appeared 2 months before the date of admission (day 0), at which time the patient visited a otolaryngology department of local hospital (Hospital A) and was prescribed amoxicillin following a diagnosis of pharyngitis. The patient's throat symptoms persisted, and abdominal pain appeared. Body temperature measurements taken at home consistently remained at approximately 37℃. Eighteen days later (day 18), the patient was seen by the gastroenterology department of Hospital A due to persistent abdominal symptoms and was prescribed a proton‐pump inhibitor. Three days later (day 21), the patient developed malaise, accompanied by chest and abdominal pain, and was transferred to another medical facility (Hospital B) via ambulance. The patient presented with a fever of 38.3℃ at the time of transfer, and the electrocardiograph, blood tests, and chest computed tomography scan yielded no noteworthy results. He was subsequently placed under observation and then released, free to return home. The patient's symptoms were found to persist after an additional week of monitoring. Although abdominal ultrasonography and urinalysis had also been performed at Hospital A, no issue was flagged. One month after the initial onset of symptoms (day 31), the patient underwent another blood test as well as an upper gastrointestinal endoscopy at another medical institution (Hospital C), but no abnormalities were detected. After another four weeks (day 58), the patient underwent an abdominal CT at Hospital C. As a result, he was diagnosed with functional dyspepsia (FD) and was prescribed Acotiamide and Bifidobacterium. From that day onwards, the patient began to experience glossodynia, difficulty consuming food, and he visited our clinic on day 59. Except for slight sign of geographic tongue, no clear intraoral pharyngeal findings were observed as a result of an examination. The SARS‐CoV‐2 PCR test was performed using a saliva sample after taking into consideration the atypical progress of symptoms and the incidence of COVID‐19 in residential areas. The test came back positive 2 days later. An antibody test (INNOVITA) administered on day 59 returned negative results for both IgM and IgG. The patient was then admitted to a community hospital for follow‐up observation and was discharged after several days without any abnormal blood test or CT scan results. No serious reoccurrence of symptoms has occurred to date.

## 3 DISCUSSION

Here, we report on a COVID‐19 case with persistent gastrointestinal symptoms. Although this was a mild case, the patient experienced sustained nonspecific symptoms and had to visit several hospitals before he was diagnosed with a novel coronavirus infection. Diagnosis of asymptomatic or mild COVID‐19 cases is essential in preventing the spread of infection. However, this case highlights the limitations of diagnosing COVID‐19 cases based only on symptom presentation during the pandemic.

To date, COVID‐19 is thought to manifest in conjunction with mild symptoms shared with other common viral infections, such as fever (85.1%‐98%),^1^, ^5^ cough (59.4%‐76%),^1^, ^5^ and malaise (31%‐69.6%).^1^, ^6^ Even in cases where the infection becomes more severe, it has been noted that it takes approximately one week to reach the level of severe respiratory distress and acute respiratory distress syndrome (ARDS). Asymptomatic infections have also been reported to make up 1.0% ‐ 17.9% of all infections.^7^, ^8^ Regardless, considering the characteristics of COVID‐19 described above, accurate diagnoses are critical for infection control, and as a result, there are few options outside of expanding the scale of testing. However, in some countries including Japan, there are places where large‐scale PCR testing is not possible.^9^ In these places, there is a question of which patients should be prioritized for testing for COVID‐19, and clinicians have devoted their efforts to estimating the likelihood of COVID‐19 infection in advance. Such a situation may stress the capacity of medical staff and infrastructure, and improvements in the diagnostic process are urgently needed.

The present case suggests the need for caution when diagnosing gastroesophageal reflux disease (GERD) or FD during the COVID‐19 pandemic. COVID‐19 has been reported to cause not just respiratory‐related symptoms, but also gastrointestinal symptoms such as diarrhea (3%‐10.1%), and vomiting and nausea (3.2%‐10.1%).^5^ Nausea, bloating, and diarrhea are also common symptoms of gastrointestinal tract infections, and it may be underestimated that patients with similar symptoms may be infected with COVID‐19.^10^ COVID‐19 has also been noted to potentially cause gastrointestinal symptoms due to the high expression of angiotensin‐converting enzyme 2 receptors in the tongue, pharynx, and gastrointestinal tract.^11^ GERD is one of the most common causes of nasopharyngeal discomfort. It affects the epigastric region, causes cough symptoms, and is seen in routine medical practice. As a result, it can be difficult to distinguish COVID‐19 from GERD due to the similar gastrointestinal symptoms. In addition, functional digestive tract diseases such as FD and irritable bowel syndrome (IBS) are known to be exacerbated by viral infections,^12^, ^13^ and it is important to remember that there is the possibility of the novel coronavirus infection even in cases of discrete pathologies. Gastrointestinal symptoms of COVID‐19 have been reported to persist for 1 ‐ 9 days,^5^ but the patient in this case study experienced symptoms for much longer than seen in past cases. Although this patient resides in an area of Japan experiencing an epidemic of COVID‐19, he had no known contact with infected persons prior to onset. The result of the PCR test was positive, but this does not necessarily prove active infection at the time of testing. Therefore, it is difficult to estimate when the patient contracted the novel coronavirus, and it is not clear which symptoms can be associated with COVID‐19. Two probable explanations can be considered: First, this patient had FD and GERD‐like symptoms as one of the symptoms of COVID‐19 infection. The other is that the FD and GERD appeared after the COVID‐19 infection. Further collection and evaluation of case data regarding the impact of novel coronavirus infection on postinfection gastrointestinal symptoms are needed.

The upper gastrointestinal tract symptoms of COVID‐19 are difficult be correctly diagnosed in a clinical setting, and underestimating the possibility of COVID‐19 infection could increase the exposure of medical personnel to the infection. While the spread of COVID‐19 has become a problem in many countries, the infection of personnel at medical facilities is also an issue.^14^ In addition to routine blood sampling and imaging tests, upper gastrointestinal tract endoscopy is often used to investigate the cause of gastrointestinal symptoms. However, there is reason to believe that it is one of the riskiest procedures, increasing the likelihood of medical personnel being exposed to novel coronavirus infection.^15^ For this reason, caution is needed when performing endoscopies during the COVID‐19 pandemic, especially in areas where the spread is particularly pronounced.

In addition to the gastrointestinal symptoms presented by the present case, it has been reported that some cases of COVID‐19 are difficult to diagnose due to atypical symptoms, including altered mental status,^16^ abdominal and testicular pain,^17^ delirium,^18^ and anosmia.^19^ These atypical symptoms sometimes precede or occur independently of respiratory symptoms and are therefore make it difficult to even suspect as COVID‐19 infection case, which is a common concern of healthcare workers involved with COVID‐19. Thus, it is important to educate medical staff, particularly those in COVID‐19 epidemic areas, that some COVID‐19 patients may not exhibit respiratory symptoms and that patients exhibiting upper gastrointestinal or other such atypical symptoms may need to be further examined as candidates for testing for a potential COVID‐19 infection.

During the COVID‐19 pandemic, promptly isolating infected people and diagnosing mild cases is necessary. The medical field is facing a host of new challenges during this pandemic, and learning how to handle mild cases, such as the case presented here, is a major issue.

## CONFLICT OF INTEREST

None declared.

## AUTHOR CONTRIBUTIONS

HS and AO: involved in study concept and design. HS and AO: drafted the manuscript. YM and. KT: involved in critical revision and supervision. All authors discussed the results and commented on the manuscript. All authors approved the contents of the submitted manuscript.

## ETHICAL APPROVAL

The written informed consent for the report of the case was obtained from the patient.
